# Treatment of Allergic Rhinitis with Ectoine Containing Nasal Spray and Eye Drops in Comparison with Azelastine Containing Nasal Spray and Eye Drops or with Cromoglycic Acid Containing Nasal Spray

**DOI:** 10.1155/2014/176597

**Published:** 2014-06-01

**Authors:** Nina Werkhäuser, Andreas Bilstein, Uwe Sonnemann

**Affiliations:** ^1^Bitop AG, Stockumer Straße 28, 58453 Witten, Germany; ^2^Private Health Centre, Institute for ENT Elmshorn, Hermann-Ehlers-Weg 4, 25337 Elmshorn, Germany

## Abstract

*Objectives*. Allergic rhinitis is a common disease with increasing prevalence and high impact on economic burden and comorbidities. As treatment with pharmacological drugs is not always satisfactory due to side effects and incomplete efficacy, alternative treatment strategies are needed. Ectoine is an osmolyte with membrane stabilizing and inflammation reducing capacities. Nasal spray and eye drops containing ectoine are promising new treatment regimens for allergic rhinitis sufferers. *Design and Methods*. The current two noninterventional trials evaluated the efficacy and safety of ectoine containing nasal spray and eye drops for treating allergic rhinitis in comparison with either azelastine or cromoglycic acid containing products. Nasal and ocular symptom developments as well as judgment of tolerability and efficacy were assessed both by investigators and patients over a time period of one to two weeks. *Results*. Both trials confirmed that ectoine containing products reduced nasal and ocular symptoms in allergic rhinitis patients. Results clearly demonstrated good safety profiles of the ectoine products comparable to those of azelastine and even better to those of cromoglycate products. *Conclusion*. Ectoine containing nasal spray and eye drops are interesting new treatment strategies for sufferers of allergic rhinitis, combining both good efficacy and absence of side effects.

## 1. Introduction

Allergic rhinitis is a common disease affecting 10–20% of the population [1]. Since it has great impact on patients' quality of life, school performance, work productivity, and comorbid conditions such as asthma, it is considered as an important health problem. Allergic rhinitis is defined as an allergic reaction (most often IgE-dependent) to offending allergens such as dust mites, insects, animal dander, and pollens. Symptoms include rhinorrhea, nasal obstruction, nasal and nasopharyngeal itching, sneezing, and postnasal drip. Often, allergic rhinitis is accompanied by allergic conjunctivitis with ocular symptoms such as itchy and watery eyes, resulting in the term allergic rhinoconjunctivitis. According to its length of duration, allergic rhinitis is classified into intermittent (symptoms present <4 days a week of <4 weeks) and persistent (symptoms present ≥ 4 days a week and for at least 4 weeks) forms. Symptom severity is used to classify allergic rhinitis into mild or moderate-severe forms.

A number of pharmacological treatments of allergic rhinitis exist, such as, for example, oral and topical antihistamines, leukotriene receptor antagonists, intranasal glucocorticoids, and cromoglycic acid (mast cell stabilizers) [2].

Azelastine is a new-generation antihistamine applied topically as nasal spray or eye drops. It is used as treatment of allergic rhinitis, hay fever, and allergic conjunctivitis. Although azelastine is regarded as effective possible first-line treatment for allergic rhinitis, common side effects, such as bitter taste of the drug and local irritation reactions and rare side effects such as fatigue or headache, can occur [3].

Cromoglycic acid is an antiallergic drug which inhibits the degranulation of mast cells, thereby blocking the release of inflammatory mediators [4]. Thus, cromoglycic acid prevents the development of allergic reactions rather than reducing acute symptoms and its onset of action is about four to seven days. Due to its short half-life, cromoglycic acid has to be applied at least 4 times a day. Cromoglycic acid is thought to be a safe medication, and adverse events which might occur are usually mild, such as sneezing and sensation of burning. Due to its good safety profile, cromoglycic acid can be prescribed for treating rhinitis in children and pregnant women.

In general, many allergic rhinitis patients are still unsatisfied with the control of symptoms, complain about incomplete relief of symptoms, and suffer from unwanted side effects [5, 6]. Therefore, it is not surprising that increasing interest in the use of alternative and complementary medicine (CAM) for treating rhinitis exists. Thus, it was demonstrated that 40% of the American population uses CAM, 17% of which uses it for treating otorhinolaryngologic diseases [7]. However, so far no general recommendation for the use of CAM can be given by ARIA guidelines as ambiguous study results are available [8].

The present two individual studies compared treatment of allergic rhinitis with ectoine containing nasal spray and eye drops with azelastine containing products (study 1) or treatment with ectoine containing nasal spray with that of cromoglycic acid containing nasal spray (study 2).

Ectoine is a natural amino acid derivate which is produced by bacteria living under extreme harsh environmental conditions where it serves as osmoregulatory compatible solute [9, 10]. Ectoine works via a mechanism called “preferential exclusion” [11, 12]. If it is present together with proteins or lipids, ectoine is expelled from their surfaces, thereby increasing the hydration of the surface and stabilizing lipid layers [13]. Its membrane stabilizing as well as inflammation reducing capacities makes ectoine an interesting candidate for the treatment of allergic rhinitis. These studies served to investigate the efficacy and safety of ectoine containing nasal spray and eye drops in patients with allergic rhinitis.

## 2. Materials and Methods

The current paper describes two noninterventional studies carried out with ectoine containing nasal spray and eye drops assessing their efficacy in comparison with azelastine nasal spray and eye drops (study 1, NCT02131051) or cromoglycic acid nasal spray (study 2, NCT02131038).

### 2.1. Medication

The ectoine eye drops contain an iso-osmotic solution with 2% ectoine and 0.35% hydroxyethyl cellulose; the ectoine nasal spray is a hypertonic solution with 2% ectoine. Additional ingredients of the eye drops were sodium chloride, sodium dihydrogen phosphate dihydrate, sodium monohydrogen phosphate dihydrate, and water. Additional ingredients of the nasal spray were sodium chloride and water. In study 1, both nasal spray and eye drops were used, whereas only the nasal spray was used in study 2.

Azelastine containing products were used as comparator in study 1. The azelastine eye drops contain 0.5 mg/mL azelastine hydrochloride with one drop administering 0.015 mg azelastine hydrochloride, and the azelastine nasal spray contains 1 mg/mL azelastine hydrochloride with one puff administering 0.14 mg azelastine hydrochloride. Additional ingredients of the eye drops were benzalkonium chloride (preservative), sodium edetate, hypromellose, sorbitol, sodium hydroxide, and water. Additional ingredients of the nasal spray were sodium edetate, hypromellose, citric acid, sodium chloride, sodium hydrogen phosphate, and water.

During study 2, a cromoglycic acid containing nasal spray was used as comparator. The spray contained 20 mg/ml cromoglycic acid corresponding to 2.8 mg sodium cromoglycic acid per puff. In addition, the following ingredients were present in the formulation: benzalkonium chloride (preservative 0.014 mg/puff), sodium edetate, sodium chloride, sodium dihydrogen phosphate, sodium monohydrogen phosphate, sorbitol, and water.

### 2.2. Treatment and Study Design

#### 2.2.1. Study 1

On day 0 (Visit 1) patients were asked to participate in the study, and upon signing the informed consent form and patient information, they were allocated to one of the study groups, without any washout period. Antiallergic medications used the last two days prior to inclusion were recorded by the physician. Patients were treated either with ectoine containing nasal spray and eye drops or with azelastine containing nasal spray and eye drops. Patients of the ectoine group had to apply one eye drop per eye and one puff of the nasal spray per nostril four times per day. Patients of the azelastine group had to apply one eye drop per eye and one puff of the nasal spray per nostril twice per day. The treatment period was 7 days, and patients were asked to document their symptoms, together with possible comedication and adverse effects daily in patient diaries at the evening. Therefore the patients' assessments started after the products had been applied already. Following treatment, patients came back for Visit 2 (day 7), during which symptom scores were evaluated and tolerability, efficacy and compliance, and possibly comedications, antiallergic and others, were assessed.

*In- and Exclusion Criteria.* Male or female patients aged 18–70 with proven allergy and acute symptoms in nose and eye (sum nasal score ≥ 15 and sum oral score ≥ 6) were allowed to take part in the study. Allergy diagnosis was based on positive prick test. Exclusion criteria were pregnant and nursing women, drug addicts and persons unable to give consent to study participation, patients with intolerance against ingredients of any of the study treatments, previous eye or nose surgery, concomitant treatment with antiallergic drugs, and diseases which might influence the output of the study according to the physicians' judgment.

*Scoring of Nasal and Ocular Symptoms.* Single nasal (nasal obstruction, rhinorrhea, and sneezing) and ocular symptoms (eye itching, tearing, and conjunctivitis) were scored with an 8 point scale ranging from no symptoms (0) to very severe symptoms (8).

*Scoring of Efficacy, Tolerability, and Compliance.* Efficacy, tolerability, and compliance were judged by using a scale ranging from 0 (very good) to 8 (bad). Thus, a general judgment, of either how well to tolerate or how efficient the products were, had to be given by the patients and documented in the patient diaries. Both scoring values were based on the patients' personal opinion/feeling with the products. Whereas efficacy and tolerability were assessed both by patients and by physicians, compliance was solely judged by physicians.

*Statistics.* The statistical analysis was carried out with SPSS version 18 and SigmaPlot version 12. Both efficacy and safety analyses were performed on the entire study population. Descriptive statistics were used for a quantitative report of the main study population features. Continuous variables were tested for normal distribution via Kolmogorov-Smirnov test. Further analysis was carried out with the Mann-Whitney *U* test, Wilcoxon test, or Friedman test. The level of significance was set to *P* < 0.05 in all tests. Unavailable data were treated as “missing values” or substituted by the “last value carried forward” method.

#### 2.2.2. Study 2

This study was designed as a crossover study, without any washout period within the first week. Half of the patients received ectoine nasal spray whereas the other half received cromoglycic acid containing nasal spray. After 7 days, patients swapped to the other treatment. Thus, patients who started with one week treatment with ectoine nasal spray received cromoglycic acid containing nasal spray within the second week and vice versa. For simplification reasons, patients starting their treatment with ectoine are termed group A, and patients starting their treatment with cromoglycic acid are termed group B in this paper.

The ectoine nasal spray had to be applied at least 5 times per day, whereas the cromoglycic acid spray had to be applied 4 times a day. Thus, patients had to take the ectoine product at least 5 times a day but could upgrade dosing if they felt that medication was not sufficient. The cromoglycic acid product had to be used according to the instruction for use.

Patients had to attend visits to the investigator on day 0 (V1), day 7 (+2 days) (V2), and day 14 (+2 days) (V3). During those visits, the investigator assessed nasal (nasal obstruction, sneezing, and rhinorrhea) and ocular symptoms (eye itching, tearing, and conjunctivitis) as well as palate itching and turbinate hyperplasia. At the end of the study (V3), efficacy, tolerability, and compliance were determined.

In addition to the investigator's assessment, patients had to document daily their ocular and nasal symptoms as well as their judgment of tolerability and efficacy in a patient diary at the evening. Based on the design the patients scoring started after the study medication had been applied.

*In- and Exclusion Criteria.* Male or female patients with diagnosed allergy and moderate to severe acute symptoms of nasal obstruction, sneezing, and rhinorrhea were allowed to take part in the study. The diagnosis of the allergy was based on a positive prick test. Exclusion criteria were intolerance against ectoine or cromoglycic acid, pregnancy, previous nose surgeries, or ongoing treatment with additional antiallergic drugs.

*Scoring of Nasal, Ocular, and Other Symptoms.* Single nasal symptoms (nasal obstruction, rhinorrhea, and sneezing) and ocular symptoms (eye itching, tearing, and conjunctivitis) as well as the symptoms palate itching and turbinate hyperplasia were scored with an 8 point scale ranging from no symptoms (0) to very severe symptoms (8). 

*Scoring of Efficacy, Tolerability, and Compliance.* Efficacy, tolerability, and compliance were judged by using a scale ranging from 0 (very good) to 8 (bad). Thus, a general broad judgment, of how well to tolerate and how efficient the products were, had to be given by the patients and to be documented in the patient diaries. Both scoring values were based on the patients' personal opinion/feeling with the products. Whereas efficacy and tolerability were assessed both by patients and by physicians, compliance was solely judged by physicians.

*Pollen Score.* In order to reflect the current pollen exposure, data from the online HEXAL pollen calendar were used to grade pollen exposure into mild, moderate, or severe (1, 2, or 3) scores during the course of the study. 

*Statistics.* The statistical analysis was carried out with SPSS version 17 and SigmaPlot version 12. Safety analyses were performed on the entire study population whereas efficacy analysis was performed on all patients who completed the treatment. Continuous variables were tested for normal distribution via Kolmogorov-Smirnov test. Further analysis was carried out with the Mann-Whitney *U* test, Wilcoxon test, or Friedman test. The level of significance was set to *P* < 0.05 in all tests. Unavailable data were treated as “missing values” or substituted by the “last value carried forward” method.

## 3. Results

Both studies were conducted in accordance with the Declaration of Helsinki. All investigations were carried out with the understanding and consent of all participants.

### 3.1. Results Study 1

**Figure 1:**
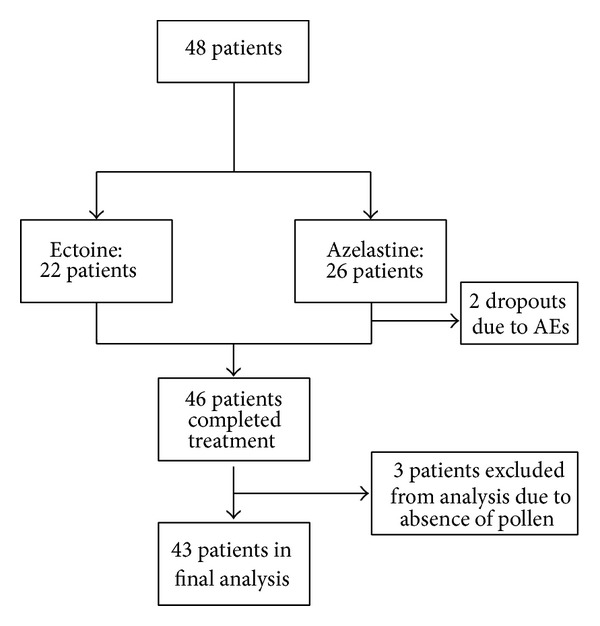
Patient flow during study 1.

This was a noninterventional trial taking place at two German ear nose throat (ENT) practices starting in June 2010 and being completed in September 2010. Distribution of patients is shown in Figure 1. In total, 48 patients took part in the study, of which 43 were included in the final analysis (31 females and 12 males). Mean age of patients was 35 years, and both groups were comparable in regard to clinical aspects.

#### 3.1.1. Nasal Symptoms

Nasal symptom scores were assessed both as single symptoms and as sum of all nasal symptoms (TNSS). Details of the development of single scores are given in Table 1. 

*Nasal Obstruction.* The mean symptom score of nasal obstruction decreased significantly by 45.95% in the ectoine group and by 49.23% in the azelastine group (V1 to V2, *P* < 0.001 for both groups). The documentation of the patient diaries also reflected a significant decrease by 18.39% in the ectoine group (*P* = 0.003) and by 17.83% in the azelastine group (*P* = 0.044).

*Rhinorrhea.* A significant decrease in the symptom score was also observed for rhinorrhea from V1 to V2. Mean values decreased by 56.88% in the ectoine group and by 52.50% in the azelastine group (*P* < 0.001 for both groups). The patient documentation showed a clear decrease of the symptom rhinorrhea which, however, was not significant. Values decreased by 28.75% in the ectoine group (*P* = 0.054) and by 19.45% in the azelastine group (*P* = 0.133).

*Sneezing.* The symptom sneezing decreased significantly from V1 to V2: values decreased by 59.52% in the ectoine group and by 59.84% in the azelastine group (*P* < 0.001 for both groups). The patient documentation also reflected the symptom decrease which was not significant in the ectoine group (25.61%, *P* = 0.475) but significant in the azelastine group (39.47%, *P* < 0.001).

*Nasal Itching.* Nasal itching decreased significantly from V1 to V2: values decreased by 76.40% in the ectoine group (*P* = 0.001) and by 69.31% in the azelastine group (*P* < 0.001). According to the patient documentation, nasal itching scores decreased by 27.12% in the ectoine group (*P* = 0.068) and by 42.38% (*P* = 0.002) in the azelastine group.

#### 3.1.2. Total Nasal Symptom Score (TNSS)

**Figure 2:**
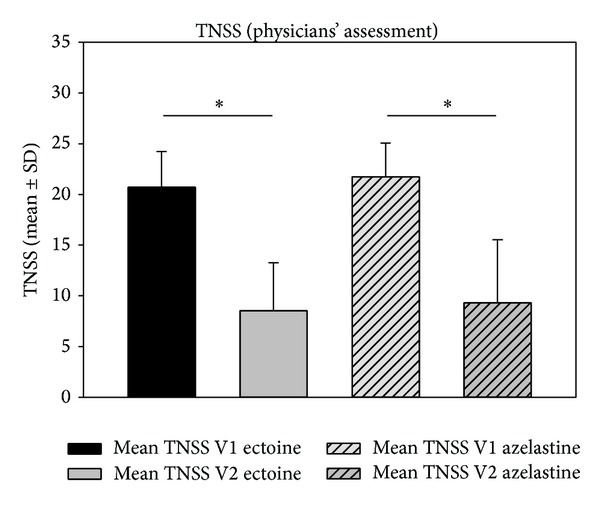
Decrease (mean ± SD) of TNSS from V1 to V2 according to the physicians' assessment. *P < 0.001.

**Figure 3:**
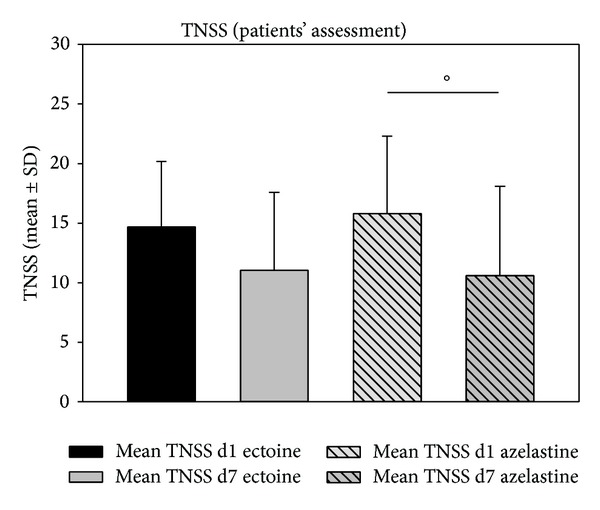
Decrease (mean ± SD) of TNSS from day 1 (d1) to day 7 (d7) according to the patients' assessment. °*P* = 0.02.

The sum of nasal symptom scores (nasal obstruction, rhinorrhea, sneezing, and nasal itching) showed a significant decrease from V1 to V2 (as assessed by physicians): sum scores in the ectoine group decreased from 20.71 ± 3.52 to 8.52 ± 4.74 (decrease of 58.85%; *P* < 0.001) and sum scores in the azelastine group decreased from 21.73 ± 3.34 to 9.32 ± 6.24 (decrease of 57.11%; *P* < 0.001). Data are depicted in Figure 2. According to the patients' assessment (see Figure 3), values decreased by 23.05% in the ectoine group (*P* = 0.076) and by 33.14% in the azelastine group (*P* = 0.02).

#### 3.1.3. Ocular Symptoms

Ocular symptom scores were also assessed as single symptoms and as sum of all ocular symptoms (TOSS). Details of the development of single scores are given in Table 2. 

*Conjunctivitis.* The symptom conjunctivitis clearly decreased from V1 to V2, as reflected by decline of 48.15% in the ectoine group (*P* = 0.058) and of 46.07% in the azelastine group (*P* = 0.013). In the patients documentation, scores of conjunctivitis decreased by 34.09% in the ectoine group (*P* = 0.218) whereas an increase by 14.77% was observed in the azelastine group (*P* = 0.885).

*Eye Itching.* There was a significant decrease in the symptom scores of eye itching: in the ectoine group, the mean decreased by 48.15% (*P* = 0.008) whereas values of the azelastine group decreased by 46.07% (*P* = 0.002). Corresponding decreases as assessed by the patients were 17.65% in the ectoine group (*P* = 0.604) and 5.33% in the azelastine group (*P* = 0.14).

*Tearing.* A statistical decrease in the scoring of the symptom tearing was also observed from V1 to V2: in the ectoine group, values decreased by 52.46% (*P* = 0.003) whereas values in the azelastine group decreased by 40.43% (*P* = 0.039). The patient documentation of the symptom tearing also showed a clear decrease of values (5.56% with *P* = 0.886 in the ectoine group and 18.63% with *P* = 0.357 in the azelastine group).

#### 3.1.4. Total Ocular Symptom Score (TOSS)

**Figure 4:**
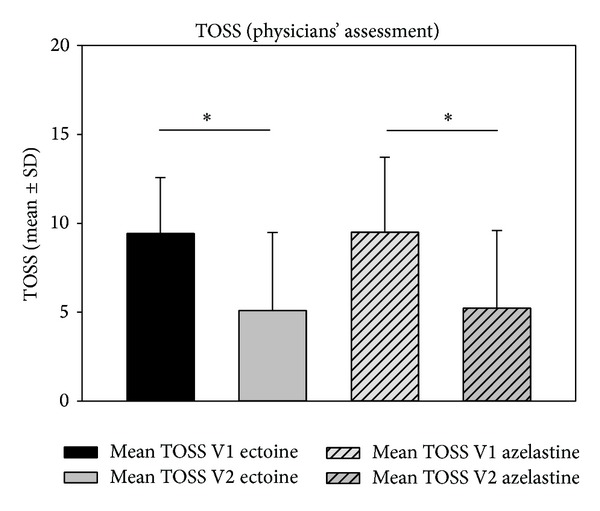
Decrease (mean ± SD) of TOSS from V1 to V2 as assessed by physicians in study 1. *P < 0.001.

The TOSS (sum of conjunctivitis, eye itching, and tearing) decreased significantly from V1 to V2 in both groups (*P* < 0.001 for ectoine, *P* = 0.009 for azelastine). Starting mean values at V1 were 9.43 ± 3.14 in the ectoine group and 9.5 ± 4.22 in the azelastine group which decreased by 45.96% to 5.10 ± 4.38 in the ectoine group and by 44.98% to 5.23 ± 4.36 in the azelastine group. Decreases of TOSS values as assessed by patients were not significant (Figure 4) (data not shown). 

*Palate Itching.* As for nasal and ocular symptoms, a clear decrease of the symptom palate itching was observed from V1 to V2: in the ectoine group, values decreased from 2.52 ± 2.71 to 1.19 ± 1.72 (*P* = 0.024), and in the azelastine group, values decreased from 3.36 ± 2.68 to 1.5 ± 1.92 (*P* = 0.018). Values of the patients' documentation did only reach statistical significance in the azelastine group: here, the scoring decreased from 3.81 ± 2.5 to 2.15 ± 2.13 (*P* < 0.001). In the ectoine group, values decreased from 1.76 ± 2.1 to 1.67 ± 2.15 (*P* = 0.854).

*Correlation of Pollen Count and Nasal Symptoms.* In order to normalize the nasal symptoms (nasal constriction, rhinorrhea, and sneezing) to the pollen burden, a quotient from sum score and pollen counts was determined. Values of quotients decreased significantly from 8.97 ± 3.98 to 5.23 ± 3.59 in the ectoine group (*P* = 0.002) and from 9.73 ± 3.59 to 5.76 ± 5.26 in the azelastine group (*P* = 0.011), thus confirming the decrease of nasal symptoms during the pollen season upon treatment.

*Efficacy, Tolerability, and Compliance.* The physicians' assessment of efficacy of both products was similar at V2, and with values of 2.48 (good) in the ectoine group and 2.64 (good-satisfactory) in the azelastine group, there was no significant difference between groups. The general tolerability was assessed as very good to good in both groups (1.33 in the ectoine group and 1.45 in the azelastine group), and the compliance was comparably good (values <1) in both groups.

**Figure 5:**
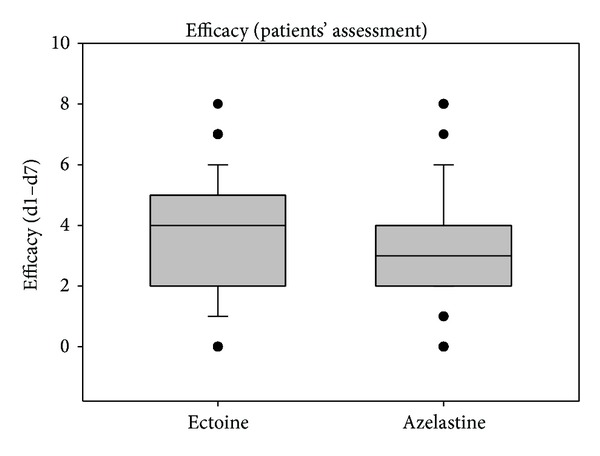
Patients' assessment of efficacy during study 1 from day 1 to day 7. Lines within the box mark the median; the upper and lower ends of the box indicate the 75th and 25th percentiles, respectively. Whiskers above and below the box indicate the 90th and 10th percentiles. Dots (•) represent outlying points.

**Figure 6:**
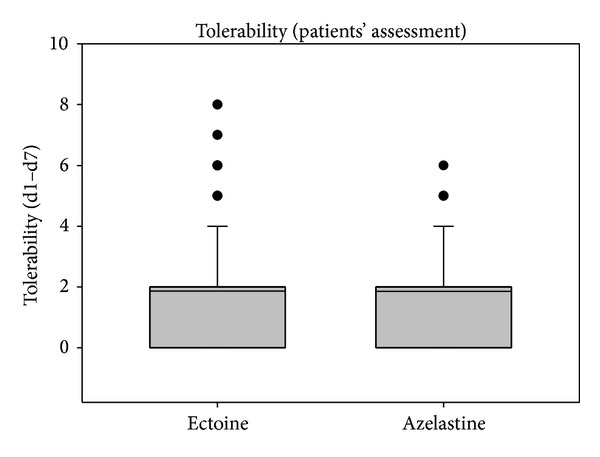
Patients' assessment of tolerability during study 1 from day 1 to day 7. Lines within the box mark the median; the upper and lower ends of the box indicate the 75th and 25th percentiles, respectively. Whiskers above the box indicate the 90th percentile. Dots (•) represent outlying points.

Values of the patients' assessments of efficacy and tolerability are shown in Figures 5 and 6. The patients' evaluations resulted in comparable values of efficacy and tolerability without statistical differences between treatment groups.

*Comparison of Reduction of Symptoms between Groups.* In order to calculate if reduction of symptoms from V1 to V2 was different between the treatment groups, differences of mean V1 and V2 values were compared via Mann-Whitney *U* test. As shown in Table 3, there were no statistical differences between the ectoine and the azelastine group, thus confirming that both substances worked comparably well. The same calculation was performed for the patient data. Here, no statistical difference was shown except for the symptom palate itching. Details are shown in Table 4.

*Adverse Events (AEs).* In total, 8 AEs occurred during the study (see Table 5). 2 AEs occurred in the ectoine group, whereas 6 AEs occurred in the azelastine group. 2 AEs in the azelastine group led to dropout of the study. No serious adverse event (SAE) occurred during the study.

### 3.2. Results Study 2

**Figure 7:**
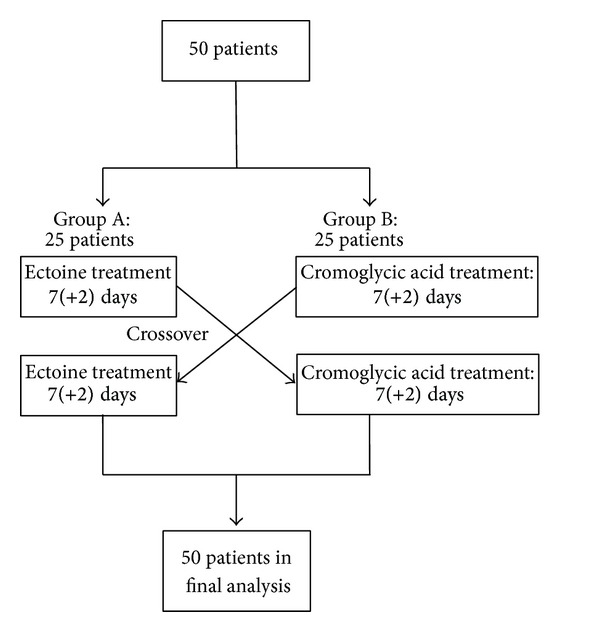
Patient flow during study 2.

This was a noninterventional trial taking place at a German ear nose throat (ENT) practice starting in May 2009 and being completed in September 2009. Distribution of patients is shown in Figure 7. In total, 50 patients (33 females and 17 males) with an average age of 34 years took part in the study. Both treatment groups were homogeneous from a clinical point of view.

#### 3.2.1. Nasal Symptoms

*Nasal Obstruction. *Both patient groups started with a comparable mean nasal obstruction score of 5.80 in group A and 5.64 in group B (physician's assessment). The symptom scores decreased to 3.2 (group A) and 3.44 (group B) after a week, and a further decrease to 2.52 (group A) and 2.92 (group B) was observed after 2 weeks. Decreases were significant in both groups with *P* values both for 1 week and for 2 weeks of *P* < 0.001.

Similarly, patient scores of the symptom nasal obstruction decreased from 4.08 (group A) and 3.60 (group B) on day 0 to 2.84 (group A, *P* = 0.009) and 3.24 (group B, *P* = 0.464) on day 7 and further to 2.52 (group A, *P* = 0.004) and 2.56 (group B, *P* = 0.041) on day 14.

*Rhinorrhea. *The symptom rhinorrhea decreased significantly (*P* < 0.001) for both groups both from V1 to V2 and from V1 to V3 according to the physician's assessment. Values decreased from 5.12 to 2.40 (V2) and further to 1.88 (V3) in group A and from 4.96 to 2.68 (V2) and to 2.76 (V3) in group B.

According to the patients' evaluation, scoring of rhinorrhea decreased from 3.12 to 2.32 (d7, *P* = 0.104) and further to 2.04 (d14, *P* = 0.010) in group A. In group B, values decrease from 3.80 to 3.08 (d7, *P* = 0.115) and further to 2.28 (d14, *P* < 0.001).

*Sneezing. *The symptom sneezing also decreased significantly (*P* < 0.001) from V1 to V2 and from V1 to V3 in both groups. Baseline scores from group A were 5.72 and decreased to 2.56 (V2) and further to 1.80 (V3), whereas values in group B decreased from 5.68 to 2.80 (V2) and to 2.6 (V3).

According to the patients' evaluation, scoring of the symptom sneezing decreased from 3.16 to 2.44 (*P* = 0.20) on day 7 to 2.12 (*P* = 0.265) on day 14 in group A, whereas values decreased from 4.04 to 2.64 (*P* = 0.018) on day 7 to 2.40 (*P* < 0.001) on day 14 in group B.

#### 3.2.2. Total Nasal Symptom Score (TNSS)

**Figure 8:**
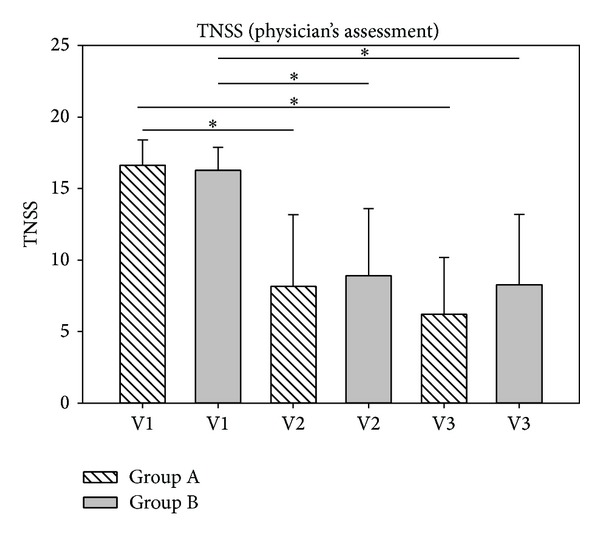
TNSS development according to the physician's assessment. TNSS scores decreased from 16.64 (V1, group A) to 8.16 (V2, group A) and further to 6.20 (V3, group A). In group B, values decreased from 16.28 (V1) to 8.92 (V2) and to 8.28 (V3). *P < 0.001.

**Figure 9:**
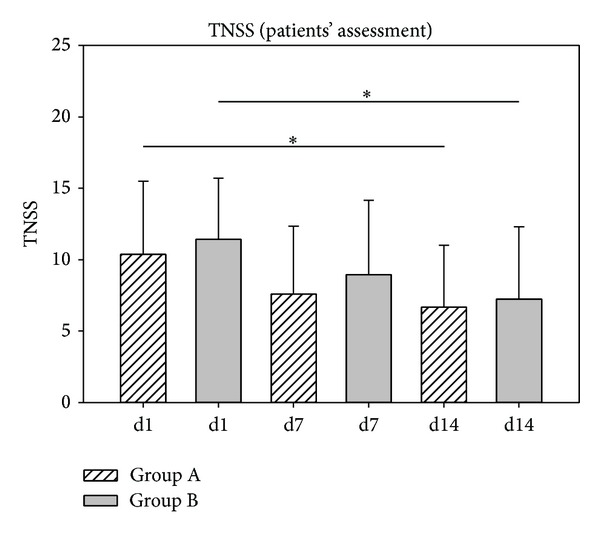
TNSS development according to the patients' assessment. TNSS scores decreased from 10.36 (d1, group A) to 7.60 (d7, group A) and further to 6.68 (d14, group A). In group B, values decreased from 11.44 (d1) to 8.96 (d7) and to 7.24 (d14). *P < 0.001.

To reflect the development of the sum of nasal symptoms, the total nasal score (nasal obstruction, rhinorrhea, and sneezing) was calculated. Results are depicted in Figures 8 and 9. According to the physician's assessment, TNSS scores decreased significantly for both groups both from V1 to V2 (*P* < 0.001) and from V1 to V3 (*P* < 0.001). Scores assessed by patients showed that decreases in TNSS from d1 to d7 were not significant whereas significant decreases in TNSS scores from d1 to d14 were shown both for group A (*P* < 0.001) and group B (*P* < 0.001).

#### 3.2.3. Ocular Symptoms

To investigate the development of ocular symptoms during the treatment period, the single symptoms eye itching, tearing, and conjunctivitis/redness of eyes were assessed both by the investigator and by the patients. Details of scores are listed in Tables 6 and 7. According to the investigator's assessment, all observed ocular symptoms improved significantly from V1 to V3 in group A, whereas only the symptoms eye itching and tearing improved significantly in group B. The patients' assessment of ocular symptoms showed that the symptoms eye itching and eye redness improved significantly in group A, whereas decreases in symptom scores from day 1 to day 14 were not significant in group B.

#### 3.2.4. Total Ocular Symptom Score (TOSS)

**Figure 10:**
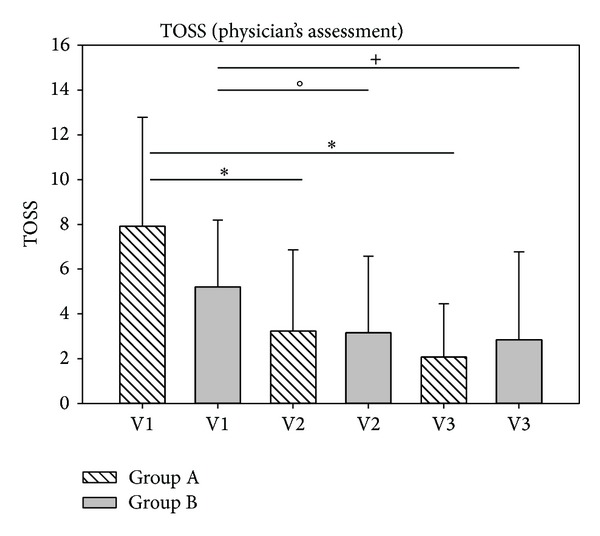
Assessment of sum of ocular symptoms (TOSS) according to physician's assessments in study 2. *P < 0.001, °*P* = 0.008, and ^+^ *P* = 0.003.

The development of the sum of ocular symptoms (eye itching, conjunctivitis, and tearing) as assessed by the investigator is depicted in Figure 10. It could be confirmed that ocular symptoms decreased significantly from V1 to V2 (*P* < 0.001 for group 1; *P* = 0.008 for group B) as well as from V1 to V3 (*P* < 0.001 for group 1; *P* = 0.003 for group B).

**Figure 11:**
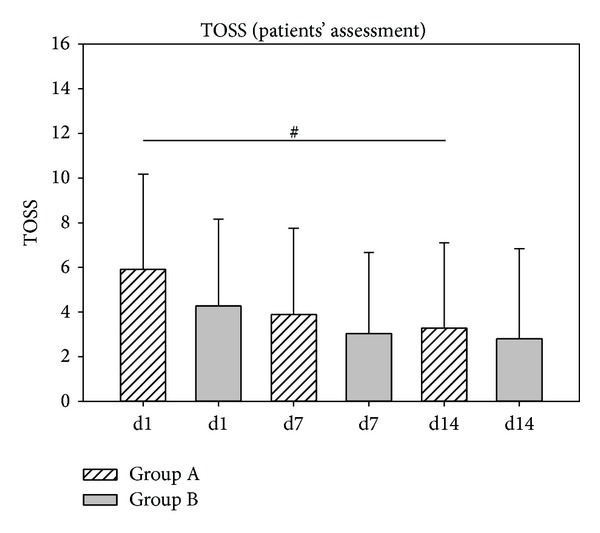
Assessment of sum of ocular symptoms TOSS development according to the patients' assessment during study 2. ^#^ *P* = 0.026.

The development of total ocular symptom score as assessed by patients is shown in Figure 11. Here, a significant decrease of symptom score was only observed in group A from day 1 to day 14 (*P* = 0.026).

*Palate Itching and Turbinate Hyperplasia.* In addition to nasal and ocular symptoms, the development of the symptom palate itching was determined both by the investigator and the patients. As shown in Table 8, significant decreases in the symptom palate itching were observed by the investigator from V1 to V3. In contrast, patients' assessment of this symptom showed only small decreases in this symptom which were not significant.

Additionally, the development of turbinate hyperplasia was determined by the investigator. As shown in Table 8, treatment resulted in a significant improvement of this symptom within the first week of treatment which was still significantly improved after two weeks of treatment. No differences between groups could be determined for this symptom (Table 9).

*Correlation of Pollen Count and Nasal Symptoms.* In order to rule out that results might be influenced by the existence of pollens, data reflecting the current pollen count were included in the analysis. The quotient of TNSS values and pollen count scores confirmed a significant decrease of TNSS values both from V1 to V2 (*P* < 0.001) and from V1 to V3 (*P* < 0.001) (data not shown).

*Efficacy, Tolerability, and Compliance.* According to the physicians' judgment, the efficacy of treatment was rated “good to satisfactory” with a score of 2.68 ± 1.89 (group A) and 2.96 ± 1.72 (group B) at V2 and a score of 3.12 ± 2.11 (group A) and 2.80 ± 2.06 (group B) at V3. There were no significant differences between both groups.

Similarly, the patients' assessment of efficacy was “good to satisfactory” both on day 7 (group A: 2.76 ± 1.89; group B: 2.96 ± 1.81) and on day 14 (group A: 2.56 ± 2.00; group B: 2.44 ± 2.16) without statistical differences between groups.

Following 1 week of treatment, the tolerability was judged as “very good” in group A (1.24 ± 1.30) and as “good” (2.40 ± 1.53) in group B. Following crossover of groups, tolerability of the treatment was judged as “satisfactory” (3.0 ± 2.16) in group A and as “very good” (0.88 ± 1.05) in group B. The changes of tolerability between both groups were highly significant (*P* < 0.001), thus indicating that tolerability was significantly better following a 7-day treatment with ectoine containing nasal spray in comparison to 7-day treatment with cromoglycic acid nasal spray.

**Figure 12:**
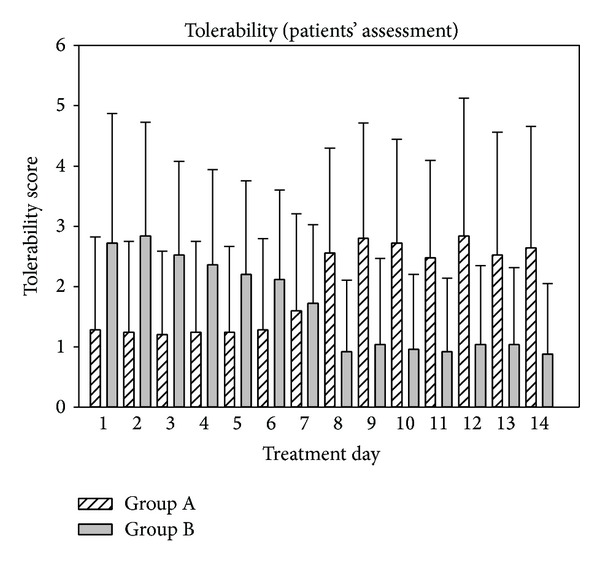
Patients' assessment of tolerability of treatments during study 2.

Those differences in tolerability scoring were also clearly visible in the patients' assessment of tolerability. Whereas tolerability was judged as “very good” (1.30 ± 1.48) within the first days of treatment in group A, scoring for the second week decreased to a mean score of 2.65 ± 1.89 corresponding to “good to satisfactory.” Group B showed the opposite development with a tolerability scoring of 2.35 ± 1.68 (“good”) within the first 7 days which improved to a mean score of 0.97 ± 1.24 (“very good”) within the second half of the treatment. Details of the patients' tolerability assessment are depicted in Figure 12. In summary, patients judged the tolerability significantly better under treatment with ectoine containing nasal spray compared to treatment with cromoglycic acid nasal spray (*P* < 0.001).

The compliance was assessed as very good by the physician, and values were not statistically different between groups (see Table 10).

*Adverse Events (AEs).* During the study, no serious adverse events (SAEs) occurred. No adverse events were observed during treatment with ectoine containing nasal spray. In contrast, 13 patients complained about a burning sensation during treatment with cromoglycic acid nasal spray. One patient complained about displeasing smell, and another patient complained about dehydration effect. The correlation between the observed AEs (burning sensation, displeasing smell, and dehydration effect) was judged as probable.

## 4. Conclusions

The current studies investigated the efficacy and safety of ectoine containing nasal spray and eye drops in comparison with commonly applied pharmacological treatments of allergic rhinitis. In two noninterventional trials, ectoine products were compared with either azelastine or cromoglycic acid containing products. Although this paper covers results of two separate studies, they were summarized in one document as the indication was very similar and both studies aimed to compare ectoine containing products with other topical medications. As the study with cromoglycic acid was one of the first studies with ectoine products, dosage was slightly higher than in the study comparing ectoine and azelastine products. As a placebo-controlled, randomized trial with ectoine nasal spray and eye drops which was conducted after the study 2 had confirmed that the dose of 4 uses per day was sufficient to show significant superiority over placebo treatment, this dosage was chosen in study 1 [14]. Both studies demonstrated that allergic rhinitis can be successfully and safely treated with ectoine containing products, thus offering a potential new treatment strategy for allergic rhinitis sufferers.

Both studies were intentionally designed as noninterventional studies based on German law. Although this study design forbids randomization of patients, use of placebo, and blinding of study medication, it still reflects the most realistic standard clinical practice. Thus, patients were included independently on their prior medication and no washout period had to be kept. In order to ensure homogeneity of patients, all had to show a certain degree of symptoms at inclusion reflected by a minimum of TNSS values. Additionally, symptom scores were correlated with pollen count scores in order to include objective measures into the analysis. Importantly, sites specialized in the area of ear nose throat practice were chosen to warrant a very precise assessment of symptoms by specialized physicians and to have a homogeneous patient population. Although we believe that valuable results can be drawn from noninterventional trials, one drawback of this study design is the fact that one cannot include a placebo group into the study population. On the other hand, it has been demonstrated that double-blind randomized placebo-controlled trials clearly have their limitations and disadvantages and that particularly patients' awareness of a placebo arm can lead to modifications of results due to patients' expectations and interpretations [15]. This was confirmed by a comparison of open and controlled study designs in neuroleptic studies, indicating that results of well performed open studies earn more attention [16].

In study 1, it was shown that both the ectoine and the azelastine products resulted in a clear decrease of symptoms of allergic rhinitis over the study period of 7 days. According to the physicians' evaluation, the symptoms nasal obstruction, rhinorrhea, sneezing, nose itching, conjunctivitis (azelastine group only), eye itching, and tearing were significantly reduced. The mean decrease of TNSS was −58.85% in the ectoine group and −57.11% in the azelastine group, thus demonstrating a strong clinical relevance. Similarly, mean decreases in TOSS were −45.96% in the ectoine group and −44.98% in the azelastine group and therewith reflect strong clinical relevance, too.

Study 2 also demonstrated a significant decrease of symptom scores upon treatment: within the first week of the study, TNSS values decreased by −50.96% (group A) and by −45.21% (group B), and decreases within the entire study period of 2 weeks were −62.74% (group A) and −49.14% (group B) according to the physician's assessment. Nasal obstruction is often caused by an enlargement of the nasal turbinates which are located on the lateral walls on each side of the nose. Thus, the significant improvement of turbinate hyperplasia as assessed by the physician underlined the efficacy of both treatments in reducing nasal obstruction.

In comparison to the physicians' assessment of symptoms, generally less strong symptom decreases were observed by patients. This might be most likely due to the fact that starting symptom values as assessed by patients themselves were lower than the physicians' values. This in turn is at least partly accredited to the fact that patients' first assessments of symptoms were documented at the end of the first treatment days whereas physicians documented baseline symptoms during the first site visit prior to the start of treatment. A recent placebo-controlled study in an environmental challenge chamber showed that 3 hours after application of the ectoine nasal spray and eye drops the symptoms were decreased by ~20%. This decrease reflects roughly the difference between the first assessment by the physicians and the first patient diary entry [14]. In addition, physicians are able to carry out ranking of symptoms based on their experience with many patients; thus, their judgment might be considered more objectively. On the other hand, symptoms such as itching of eyes, nose, and palate cannot be measured with a scientifically valid method and are thus prone to personal perception and difficult to be assessed by physicians together with patients. Taken together, an overestimation by the physician or an underestimation by the patients is not likely.

In study 1, the patients' assessment showed that for the azelastine group, decreases were significant for the symptoms nasal obstruction, sneezing, and nasal itching. For the ectoine group, values decreased significantly in the symptom nasal obstruction, whereas a clear but not significant decrease in the symptoms nasal itching, sneezing, and rhinorrhea was observed. In total, decreases of TNSS were −24.68% in the ectoine group and −35.26% in the azelastine group, thus confirming a clinical relevance of the treatment. Clear decreases in the ocular symptoms tearing and eye itching were assessed by patients of both groups; however, values did not reach significance. The symptom conjunctivitis was clearly (but not significantly) decreased in the ectoine group, whereas it became slightly worse in the azelastine group. In total, TOSS as assessed by patients decreased by −19.57% in the ectoine group and by −11.81% in the azelastine group.

Although no ocular treatment was applied in study 2, ocular symptoms of allergic rhinitis clearly improved upon treatment with ectoine or cromoglycic acid nasal spray. According to the physicians' assessment, TOSS values decreased by −59.09% in group A and by −39.32% in group B after 1 week of treatment and by −73.74% in group A and by −45.47% in group B after 2 weeks of treatment. It is not surprising that local and nonpharmaceutical nasal treatment might also influence ocular symptoms as recent studies suggest a crosstalk between the nose and eyes. The mechanism of the influence of symptoms via the nasolacrimal duct is not fully understood but thought to be via a mucosal connection, possibly via a nose-eye reflex.

The patients' assessment of ocular symptom development in study 2 confirmed a significant decrease in the symptom eye itching after two weeks' treatment in both groups. A significant reduction of the symptom eye redness was observed in group A but not in group B. However, as ocular symptom scores were generally rather small in this study and treatment aimed mainly to reduce nasal symptoms, further studies will be needed to evaluate the efficacy of treatments on ocular symptoms. Interestingly, another published study with azelastine eye drops, cromoglycate eye drops, or placebo eye drops showed superiority of both active treatments versus placebo without significant differences between the two active treatments [17], and a future study comparing treatment with ectoine eye drops only with other pharmacological eye drop formulations would be desirable.

The current studies both showed that ectoine nasal spray and eye drops can safely be applied in patients with allergic rhinitis. Patients judged the tolerability of the products as similarly good as the azelastine products and significantly better than cromoglycic acid nasal spray, and the very low numbers of AEs reflected a very good safety profile of the used treatments.

The crossover design of study 2 bears difficulties as no washout period between the crossover was carried out. However, as symptoms were assessed on a daily basis, effects following one treatment only (after one week) can be analyzed separately from the results following two treatments. As clear improvements of symptoms were already observed after one week of treatment, this time span seems sufficient to evaluate effects of either treatment A or B.

As the efficacy and safety of azelastine has been studied in a huge range of clinical trials during the last decades, one can use historical data to bring the results of the current study into a broader context (for results from comparator studies see Table 11). In addition, results from placebo groups of comparator studies can be used in order to rank the current results. Thus, comparable data confirmed a superiority of azelastine versus placebo treatment, and values indicate that effects of azelastine are usually about 2-3-fold higher than placebo. However, comparing the actual values of the current study with other studies is rather difficult as design (e.g., randomized versus nonrandomized, placebo-controlled versus not placebo-controlled, differences in length of treatment, and differences in scaling of symptoms) and dose (azelastine concentration and number of daily applications) of available studies differs enormously. In addition, most studies used nasal spray only and assessed solely nasal symptoms, whereas the current study is one of few studies acknowledging also ocular symptoms of allergic rhinitis. Taken together, results of the current study 1 showed that effects of ectoine containing products are almost comparable with those of azelastine, a well-studied drug, which has a proven superiority to placebo treatment and is commonly prescribed against allergic rhinitis.

Cromoglycic acid has been a common drug in treating allergic rhinitis, and although it is thought not to be as effective as intranasal steroids or antihistamines, it has been shown to reduce both nasal and ocular symptoms and it is therefore a reasonable therapy option. In particular, its good safety profile makes it an interesting treatment option both for children and pregnant women. As no published studies were identical with the current study design (study 2) in terms of treatment duration and analysis of end points, only general comparisons to cromoglycic acid studies can be drawn. Several studies have confirmed that cromoglycic acid is superior to placebo in patients with allergic rhinitis [24]. Thus, Schuller and colleagues [25] investigated the efficacy of Cromolyn sodium in comparison to nedocromil sodium and placebo. Over a treatment period of 8 weeks, it was demonstrated that Cromolyn resulted in a clear improvement of nasal symptoms, particularly in the symptom “stuffy nose.” A further placebo-controlled study confirmed that cromoglycate acid nasal spray provided significant relief of nasal symptoms within 2 weeks of treatments which were significant for the symptoms sneezing and nasal congestion and clearly visible but not significant for the symptoms rhinorrhea and nasal pruritus [26].

Taken together, ectoine containing nasal spray and eye drops have been demonstrated to be promising alternatives to pharmacological drugs with both good efficacy and a very good safety profile. As the ectoine nasal spray and eye drops act purely physically on the nasal and ocular mucosa, it makes those products particularly interesting for patients with reservations about pharmacological therapy. An additional study in children and adolescents with seasonal allergic rhinitis (data not yet published) has confirmed the safety of ectoine containing nasal spray and eye drops in the pediatric population. Further studies should be undertaken to further investigate the onset of action and compare it to commonly applied pharmacological drugs. Quick relief of symptoms is crucial for patients and understood to be advantageous when comparing, for example, azelastine with intranasal corticosteroids [27] and should therefore be assessed for the ectoine products. A controlled, ramdomised study which was carried out using a controlled environmental exposure chamber showed a quick onset of action of ectoine nasal spray and eye drops and confirmed the efficacy in reducing both nasal and ocular symptoms [14]. Additional studies applying the ectoine eye drops only are needed to further elucidate their impact on ocular symptoms during allergic rhinitis.

## Figures and Tables

**Table 1:** Development of single nasal scores (mean ± SD) during study 1 according to patients' and investigators' assessments.

Symptom	Group	Score d1 (patient)	Score d7 (patient)	*P* value	Score V1 (investigator)	Score V2 (investigator)	*P* value
Nasal obstruction	Ectoine	4.14 ± 1.93	3.38 ± 2.20	*P* = 0.003	5.29 ± 1.15	2.86 ± 1.49	*P* < 0.001
	Azelastine	4.38 ± 2.38	3.60 ± 2.37	*P* = 0.044	5.91 ± 1.23	3.0 ± 2.13	*P* < 0.001

Rhinorrhea	Ectoine	3.81 ± 1.86	2.71 ± 1.87	*P* = 0.054	5.19 ± 1.03	2.24 ± 1.58	*P* < 0.001
	Azelastine	3.48 ± 2.11	2.8 ± 2.02	*P* = 0.133	5.45 ± 1.01	2.59 ± 1.89	*P* < 0.001

Sneezing	Ectoine	3.9 ± 1.92	2.9 ± 1.73	*P* = 0.475	6.0 ± 1.48	2.43 ± 1.58	*P* < 0.001
	Azelastine	4.05 ± 1.43	2.45 ± 1.7	*P* < 0.001	5.77 ± 0.92	2.32 ± 2.10	*P* < 0.001

Nasal itching	Ectoine	2.81 ± 1.83	2.05 ± 1.56	*P* = 0.068	4.24 ± 2.32	1.00 ± 1.41	*P* = 0.001
	Azelastine	3.90 ± 1.70	2.25 ± 1.92	*P* = 0.002	4.59 ± 1.99	1.41 ± 1.05	*P* < 0.001

**Table 2:** Development of single ocular symptom scores during study 1 according to patients and investigators' assessments.

Symptom	Group	Score d1 (patient)	Score d7 (patient)	*P* value	Score V1 (investigator)	Score V2 (investigator)	*P* value
Conjunctivitis	Ectoine	2.1 ± 1.84	1.38 ± 1.56	*P* = 0.218	2.67 ± 0.97	1.71 ± 1.62	*P* = 0.058
	Azelastine	2.05 ± 1.77	2.35 ± 2.32	*P* = 0.885	3.32 ± 1.73	1.77 ± 1.66	*P* = 0.013

Eye itching	Ectoine	3.24 ± 1.89	2.67 ± 1.91	*P* = 0.604	3.86 ± 1.93	2.0 ± 1.79	*P* = 0.008
	Azelastine	2.9 ± 1.81	2.75 ± 2.1	*P* = 0.14	4.05 ± 1.89	2.18 ± 2.17	*P* = 0.002

Tearing	Ectoine	1.71 ± 1.35	1.62 ± 1.63	*P* = 0.886	2.90 ± 1.3	1.38 ± 1.69	*P* = 0.003
	Azelastine	1.9 ± 1.84	1.55 ± 1.67	*P* = 0.357	2.14 ± 1.67	1.27 ± 1.67	*P* = 0.039

**Table 3:** Correlation of differences of single symptoms (mean values) between V1 and V2 (based on physicians' evaluations) in study 1.

Symptom	Difference means V1-V2 ectoine	Difference means V1-V2 azelastine	*P* value
Nasal obstruction	2.43	2.91	0.546
Rhinorrhea	2.95	2.86	0.882
Sneezing	3.57	3.45	0.787
Nasal itching	3.24	3.18	0.768
Conjunctivitis	0.96	1.55	0.409
Eye itching	1.86	1.87	0.863
Tearing	1.52	0.87	0.254
Palate itching	1.33	1.86	0.426

**Table 4:** Correlation of differences of single symptoms (mean values) between d1 and d7 (based on patients' evaluations) in study 1.

Symptom	Difference means d1–d7 ectoine	Difference means d1–d7 azelastine	*P* value
Nasal obstruction	0.76	0.78	0.814
Rhinorrhea	1.1	0.68	0.446
Sneezing	1.0	1.6	0.54
Nasal itching	0.76	1.65	0.184
Conjunctivitis	0.72	−0.3	0.42
Eye itching	0.57	0.15	0.73
Tearing	0.09	0.35	0.826
Palate itching	0.09	1.66	0.034

**Table 5:** Adverse events during study 1.

	*N*	Description	Outcome
Ectoine group	2	#1: burning of eyes #2: itching of throat during application of products	Recovered

Azelastine group	6	#1–4: burning of eyes (*n* = 4)	1 premature determination of study due to AE
		#5: nausea #6 headache (*n* = 1)	1 premature determination of study due to AE

**Table 6:** Development of ocular rhinitis symptoms (mean values) during study 2 (physician's assessment).

Symptom	Group	Mean V1	Mean V2	Mean V3	*P* value V1 versus V2	*P* value V2 versus V3
Eye itching	A	3.80 ± 2.29	1.68 ± 1.84	1.00 ± 1.38	*P* < 0.001	*P* < 0.001
	B	2.72 ± 2.05	1.60 ± 1.63	1.48 ± 1.78	0.046	0.003

Tearing	A	2.32 ± 1.95	0.76 ± 1.13	0.68 ± 0.95	0.002	*P* < 0.001
	B	1.32 ± 1.35	0.84 ± 1.37	0.64 ± 1.35	0.107	0.006

Conjunctivitis	A	1.80 ± 2.06	0.80 ± 1.19	0.40 ± 0.58	0.086	0.011
	B	1.12 ± 1.13	0.75 ± 1.22	0.72 ± 1.21	0.094	0.383

**Table 7:** Development of ocular rhinitis symptoms (mean values) during study 2 (patients' assessment).

Symptom	Group	Mean d1	Mean d7	Mean d14	*P* value d1 versus d7	*P* value d1 versus d14
Eye itching	A	2.68 ± 1.99	1.56 ± 1.58	1.48 ± 1.81	0.044	0.019
	B	2.04 ± 1.93	1.48 ± 1.61	1.28 ± 1.74	0.250	0.014

Tearing	A	1.24 ± 1.36	1.20 ± 1.26	0.92 ± 1.08	0.992	0.382
	B	1.12 ± 1.36	1.30 ± 1.24	0.68 ± 1.41	0.271	0.297

Eye redness	A	2.00 ± 2.02	1.12 ± 1.24	0.88 ± 1.20	0.150	0.003
	B	1.12 ± 1.42	0.80 ± 1.32	0.84 ± 1.31	0.337	0.292

**Table 8:** Physician's assessment of palate itching and turbinate hyperplasia during study 2.

Symptom	Group	Mean V1	Mean V2	Mean V3	*P* value V1 versus V2	*P* value V1 versus V3
Palate itching	A	2.32 ± 2.54	1.12 ± 2.05	0.84 ± 1.52	0.054	0.003
	B	2.20 ± 2.36	1.24 ± 2.13	0.92 ± 1.80	0.048	0.002

Turbinate hyperplasia	A	4.68 ± 1.25	3.56 ± 1.08	3.76 ± 1.69	<0.001	0.007
	B	4.80 ± 1.15	3.84 ± 1.25	3.56 ± 1.19	0.005	0.001

**Table 9:** Patients' assessment of palate itching during study 2.

Symptom	Group	Mean d1	Mean d7	Mean d14	*P* value d1 versus d7	*P* value d1 versus d14
Palate itching	A	1.80 ± 2.35	1.56 ± 1.87	1.12 ± 1.62	0.962	0.425
	B	1.56 ± 1.89	1.24 ± 2.09	0.92 ± 1.78	0.053	0.035

**Table 10:** Compliance scores (assessed by the investigator) following 1 week (V2) and 2 weeks (V3) of treatment during study 2.

Group	Mean V2	Mean V3
A	1.28 ± 1.24	1.12 ± 0.97
B	0.88 ± 0.93	1.16 ± 1.03

**Table 11:** Comparison of the current study 1 with other azelastine studies.

Study	Treatment	% improvement from baseline to end of treatment	
		TNSS	TOSS
Current study 1^1^	Ectoine	58.85	45.96
	Azelastine	57.11	44.98

Current study 1^2^	Ectoine	24.68	19.57
	Azelastine	35.26	11.81

Lumry et al. 2007 (study 1)^3^ [18]	Azelastine	14.1	n.d.
	Placebo	4.5	n.d.

Lumry et al. 2007 (study 2)^3^ [18]	Azelastine	22.1	n.d.
	Placebo	12.0	n.d.

Shah et al.^4^ [19]	Azelastine 0.1%	24.4	n.d.
	Azelastine 0.15%	29.7	n.d.
	Placebo	12.0	n.d.

Howland et al.^5^ [20]	Azelastine 0.15%	19.3	16.7
	Placebo	11.4	6.0

Van Bavel et al.^6^ [21]	Azelastine 0.15%	18.7	n.d.
	Placebo	10.5	n.d.

Falser et al.^7^ [22]	Azelastine	83.56*	n.d.
	Levocabastine	70.42*	n.d.

Charpin et al.^8^ [23]	Azelastine	60.2	65.0
	Cetirizine (tablet)	63.3	60.8

^1^Physicians assessment; ^2^patients' assessment; ^3^one spray of 0.1% azelastine nasal spray per nostril twice daily for 14 days; ^4^two sprays of 0.1% or 0.15% azelastine nasal spray per nostril twice daily for 14 days; ^5^two sprays of 0.15% azelastine nasal spray per nostril once daily for 14 days; ^6^two sprays of 0.15% azelastine nasal spray per nostril once daily for 2 weeks; ^7^azelastine 1.12 mg/day and levocabastine 0.4 mg/day nasal spray administered twice daily for 4 weeks, *TNSS: sneezing, nasal itching, and rhinorrhoea; ^8^one spray of 0.1% azelastine nasal spray per nostril twice daily for 14 days. n.d.: not determined.
